# Regulation of calcium delivery and pore structure via osteoinductive microspheres of HA/CS/PGCL for orbital bone regeneration

**DOI:** 10.1093/rb/rbag001

**Published:** 2026-01-16

**Authors:** Xiaosong Zhou, Zongliang Wang, Yu Wang, Min Guo, Peibiao Zhang

**Affiliations:** Laboratory of Polymer Materials Engineering, Changchun Institute of Applied Chemistry, Chinese Academy of Sciences, Changchun 130022, P. R. China; School of Applied Chemistry and Engineering, University of Science and Technology of China, Hefei, Anhui 230026, P. R. China; Laboratory of Polymer Materials Engineering, Changchun Institute of Applied Chemistry, Chinese Academy of Sciences, Changchun 130022, P. R. China; Laboratory of Polymer Materials Engineering, Changchun Institute of Applied Chemistry, Chinese Academy of Sciences, Changchun 130022, P. R. China; Laboratory of Polymer Materials Engineering, Changchun Institute of Applied Chemistry, Chinese Academy of Sciences, Changchun 130022, P. R. China; Laboratory of Polymer Materials Engineering, Changchun Institute of Applied Chemistry, Chinese Academy of Sciences, Changchun 130022, P. R. China; School of Applied Chemistry and Engineering, University of Science and Technology of China, Hefei, Anhui 230026, P. R. China

**Keywords:** calcium delivery, hydroxyapatite, spherical calcium sulfate mesocrystals, orbital bone reconstruction

## Abstract

Repairing orbital bone defects remains a complex challenge in craniofacial surgery. Existing bone repair materials are plagued by issues such as intricate preparation processes and inadequate consideration of the spatiotemporal characteristics of bone healing. This study aimed to enhance the osteoinductive activity of poly (glycolide-co-caprolactone) (PGCL)-based materials through bicomponent modification, thereby providing a basis for the design of clinical bone repair materials. Hydroxyapatite/calcium sulfate/PGCL (HA/CS/PGCL) composite microspheres were fabricated via electrostatically assisted spray evaporation (ESASE). Their physicochemical properties and *in vitro/in vivo* osteogenic efficacy were evaluated using degradation-mineralization assays, MC3T3-E1 cell differentiation assessments and a rabbit orbital bone defect model. Innovatively, CS mesocrystal pore structure regulation was introduced to synergize with HA, achieving multidimensional optimization of surface hydrophilicity, sequential calcium ion release and mechanical properties. Results showed that *in vitro* mineralization formed native-like hydroxyapatite structures within 14 days. On HA/CS/PGCL surfaces, the proliferation activity of MC3T3-E1 cells reached 1.8 times that of the PGCL group after 7 days, with a 1.6-fold increase in alkaline phosphatase activity. *In vivo*, the composite microspheres achieved a new bone volume ratio (BV/TV) of 78.72% at 10 weeks, resulting in complete closure of the bone defect. Their trabecular structural parameters (trabecular thickness, Tb.Th; trabecular number, Tb.N) outperformed those of the single-component groups. Histological staining confirmed their ability to induce ordered collagen arrangement and the formation of dense new bone. In conclusion, the HA/CS synergistic modification strategy achieved the sequential release of calcium ions during bone repair and spatial regulation of the microsphere surface pore structure. The superior performance of HA/CS/PGCL microspheres in repairing complex bone defects lays an experimental foundation for clinical translation. Further research into the interactions between ion release and cell signaling is warranted.

## Graphical Abstract

**Figure:**
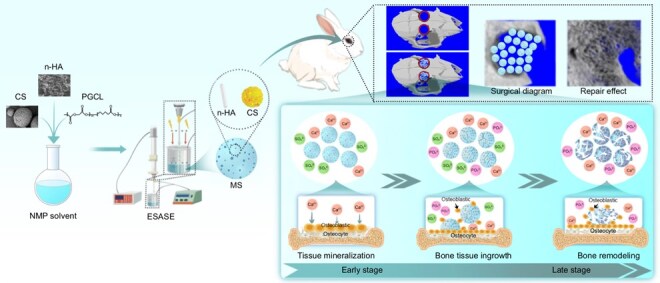

## Introduction

Orbital bone defect repair remains a complex challenge in craniofacial surgery [1]. Due to the intricate anatomical structures of the orbit, trauma, tumor resection or congenital malformations often cause issues like abnormal eye position, visual impairment and facial deformities, imposing stricter demands on bone repair material performance [2, 3]. Ideal orbital bone implant materials must closely match the morphology of the patient’s bone defect to effectively support bone tissue regeneration and repair [4, 5]. To address the complexity of processing existing materials, microspheres have emerged as a new direction due to their flexibility in adapting to various bone defect shapes, especially irregular ones [6]. Besides, their high specific surface area enhances bone bridging ability, facilitates bone tissue growth and repair and provides more sites for cell adhesion [7]. Additionally, microspheres can create a three-dimensional environment that better simulates the *in vivo* physiological environment, promoting cell adhesion, growth and differentiation while improving cell culture efficiency. The voids between microspheres enable uniform transport of nutrients and metabolites, creating favorable conditions for cell growth and metabolism, thereby advancing bone tissue repair and regeneration [8, 9]. The electrohydrodynamic spraying and solidification actuated by solvent extraction (ESASE) technique, The ESASE technology, which enables the rapid preparation of microspheres and precise control over their morphology and particle size distribution, has become a promising method for fabricating tissue engineering scaffolds [10–12].

Autologous bone grafting has long been the clinical gold standard for bone repair but is severely limited by donor scarcity [13]. Therefore, bone repair based on tissue engineering scaffolds has attracted extensive attention. An ideal bone tissue engineering scaffold should possess excellent biocompatibility, controllable degradation properties and high-efficiency osteogenic induction activity [14–16]. Various implantable biomaterials have been reported for reconstructing complex orbital bone defects. For example, Wang repaired orbital wall defects by combining bone marrow stromal cells isolated via red blood cell lysis with β-tricalcium phosphate (β-TCP) to construct grafts [17], while Peng *et al*. promoted orbital bone regeneration using low-heat bioactive glass-modified 3D-printed bioceramic scaffolds [3]. However, these commonly used implants often require additional processing to suit bone defect repair needs, with relatively complex preparation processes involving high time and economic costs. Moreover, bone regeneration is spatiotemporally complex [18]. Most bone repair materials only focus on spatial structure design while neglecting the temporal complexity of bone development, failing to respond to or even hindering the bone healing process [19]. Thus, designing bone repair materials with adaptive spatial structures that can address the temporal complexity of bone healing is key to achieving high-quality bone regeneration. Dynamic regulation of calcium ions (Ca^2+^) is critical for solving this spatiotemporal challenge. As a key element maintaining normal physiological functions, Ca^2+^ levels significantly affect bone development [20]. Studies have shown that Ca^2+^ regulates osteoblast adhesion and differentiation through calcium signaling pathways [21] and plays a key role in osteocyte differentiation and new bone mineralization [22, 23]. Incorporating Ca^2+^ into bone repair materials can, thus, significantly enhance osteogenic performance. Furthermore, Ca^2+^ acts throughout the bone healing cycle: initiating immune regulation, cell recruitment and initial collagen mineralization in the early stage; supporting osteogenic differentiation and matrix mineralization in the middle stage; and maintaining long-term mineralization and promoting woven-to-mature bone transformation in the late stage [24, 25]. Therefore, developing bone repair materials with adaptive spatial structures responsive to the temporal complexity of bone healing is essential for high-quality bone regeneration. Hydroxyapatite (HA), the main inorganic component of bone tissue, can induce mineralization by releasing Ca^2+^ and PO_4_³^-^ ions [26]. However, its slow degradation rate makes it difficult to match the calcium ion release rate with the bone repair process [27–29]. Calcium sulfate (CS), a clinical bone substitute material with good biodegradability and biocompatibility, has been widely used in bone repair [30, 31]. The ability of CS to gradually dissolve in the physiological environment and release Ca^2+^ and ${\text{SO}}_{4}^{2-}$ supports local osteogenesis, making it a preferred choice for filling bone defects caused by trauma, tumor resection or cystic lesions [30,32]. Nevertheless, the rapid degradation of CS limits its long-term clinical performance: premature dissolution may fail to provide sustained mechanical support during critical bone healing periods, leading to inadequate bone formation and compromising repair success [33, 34]. Calcium sulfate mesocrystals are hierarchical structures formed by ordered arrangement of hemihydrate calcium sulfate/bassanite nanoparticles, combining nanoparticle characteristics with macroscopic crystal periodicity [35, 36]. In ethylene glycol-water systems containing disodium ethylenediaminetetraacetate (Na_2_EDTA), irregular nanoparticles transform into rod-shaped subunits via sub-microcrystal self-assembly and directional growth, gradually forming highly ordered spherical calcium sulfate mesocrystals from outside to inside [37]. However, the application of spherical calcium sulfate mesocrystals in bone tissue engineering remains unexplored. This study aims to utilize rapid degradation of CS to enable *in situ* pore formation in mesocrystalline CS microspheres, increasing material porosity to facilitate nutrient and metabolic waste transfer at repair sites. Meanwhile, leveraging the differing degradation rates of HA (slow release) and CS (rapid release), their combination modulates material Ca^2+^ release which providing long-term calcium supply for bone repair and meeting spatiotemporal regulation requirements. Poly (ethylene glycol-co-caprolactone) (PGCL) has gained attention in bone repair for its excellent processability, adjustable elasticity, controllable degradation and good biocompatibility [38–40]. However, its hydrophobic surface, biological inertness and low osteoinductive activity limit cell adhesion and new bone formation [41, 42]. Thus, combining HA and CS with PGCL compensates for individual limitations, fabricating degradable bone repair scaffolds with high osteogenic activity.

In this study, HA/CS/PGCL composite microspheres were prepared using the ESASE technique. It also enables simple blending of HA and CS, significantly reducing the time and economic costs of material processing. CS serves not only as a calcium source but also regulates material pore formation through its mesocrystalline structure. Combining HA and CS with PGCL achieves synergistic enhancement of material properties. This study systematically evaluated the mechanical properties, hydrophilicity and *in vitro* degradation-mineralization behavior of the composite microspheres. Furthermore, their biocompatibility was verified through *in vitro* culture of MC3T3 cells. To investigate their osteoinductive properties, alkaline phosphatase (ALP) assays and gene expression analyses were performed on cells grown on the materials. The bone repair efficacy was evaluated using an *in vivo* rabbit orbital bone defect model, aiming to validate the role of dual-component modification in enhancing the osteoinductive activity of PGCL-based materials and providing experimental evidence for optimizing clinical bone defect repair materials.

## Experimental and methods

### Materials

PGCL (glycolide/caprolactone ratio is 1: 9, Mw = 80 000) and HA were purchased from Sino-tech Healthy (Jilin, China). Anhydrous calcium chloride (CaCl_2_) and anhydrous ammonium sulfate((NH_4_)_2_SO_4_) were purchased from Xilong Scientific Co., Ltd (China). Ethylenediaminetetraacetic acid disodium salt dihydrate (Na_2_EDTA·2H_2_O) purchased from Beijing chemical works. All chemicals were of analytical grade or higher. Simulated body fluids (SBF), Fetal bovine serum (FBS) and Dulbecco’s modified Eagle’s medium (DMEM) were purchased from Gibco (New York, USA). Penicillin and streptomycin were obtained from Solarbio (China). Cell Counting Kit-8 (CCK-8) was purchased from Dojindo Molecular Technologies, Inc. N-methyl pyrrolidone (NMP), Calcein-AM and propidium iodide (PI) were purchased from Aladdin (China). Polyformaldehyde (PFA) were purchased from Macklin (China). Alkaline phosphatase (ALP) assay kits were obtained from Beyotime (China), TRIzol Reagent were obtained from Invitrogen (Thermo Fisher, USA). PrimeScript RT Reagent Kit with the gDNA Eraser RR047A and SYBR Premix Ex Taq RR420A were purchased from TaKaRa (Japan). Mouse pre-osteoblast MC3T3-E1 cells were purchased from Institute of Biochemistry and Cell Biology (Shanghai Institutes for Biological Sciences, China).

### Fabrication of spherical CS mesocrystals

Spherical CS mesocrystals were prepared by the method described in the literature [43]. Monodisperse spherical CS mesocrystals were synthesized by mixing 40 mM CaCl_2_ and (NH_4_)_2_SO_4_ solution in the presence of 10 mM Na_2_EDTA·2H_2_O at 95°C and the volume ratio of ethylene glycol to water (G/W) is 5.0. After the reaction for 20 min, the resulting suspension was subjected to suction filtration, followed by three washes with boiling water and three subsequent washes with anhydrous ethanol. The obtained solid was dried under vacuum at room temperature. The dried sample was then subjected to subsequent characterization and research.

### Characterization of spherical CS mesocrystals

The X-ray diffraction (XRD) data from 10° to 90° was performed to confirm the crystal structure of CS using a D8 Advance diffractometer (Bruker Co, Germany). The TGA was obtained by heating from room temperature to 800°C in an N_2_ atmosphere. The morphology of spherical CS mesocrystals were observed by scanning electron microscopy (SEM; Philips XL30 ESEM FEG, Japan) and the elemental composition of the samples was determined by energy-dispersive X-ray energy spectrometry (EDX; Philips, XL-30 W/TMP, Japan). The porosity of the spherical CS mesocrystals was tested using a nitrogen isothermal adsorption-desorption experiment (Quantachrome Instruments; Autosorb iQ, USA).

### Preparation of microspheres

Microspheres were fabricated using the ESASE method. The custom-built laboratory apparatus consists of five key components: a support frame, an extruder, a high-voltage electrostatic (HVE) generator, a stepper motor controller and a collection device containing collection solution. A 2 mL standard plastic syringe with a 19-gauge metal nozzle is mounted on the frame, regulated by the extruder and stepper motor controller. During operation, the HVE generator applies a high voltage between the needle (positive electrode) and collection device (negative electrode). The extruder drives the polymer solution to the needle tip, where droplets form under electrostatic and gravitational forces and fall into the collection device. Solvent rapidly diffuses into the collection solution, inducing rapid polymer crystallization and solidification, enabling efficient microsphere fabrication.

For PGCL-based microspheres, PGCL was dissolved in NMP at 10% (w/v). HA and CS were then dispersed into the PGCL-NMP solution using a homogenizer to form suspensions, with feed ratios detailed in Table 1. The material groups were designated as PGCL, HA/PGCL, CS/PGCL and HA/CS/PGCL. The suspension was electro-jetted into a 4°C 75% ethanol aqueous solution in the collection device, with a 5 cm nozzle-to-device distance and 7.0 kV applied voltage. The suspension was loaded into a 2 mL syringe, and the extruder maintained an extrusion speed of 0.05 cm·min^−1^. Collected microspheres were washed thrice with excess distilled water, soaked in distilled water for 8 h to remove residual NMP and lyophilized for 12 h for subsequent analysis. This method successfully produced PGCL, HA/PGCL, CS/PGCL and HA/CS/PGCL microspheres.

**Table 1:** The feeding ratio of PGCL material in mixing.

	PGCL	HA/PGCL	CS/PGCL	HA/CS/PGCL
PGCL(g)	1	0.9	0.9	0.9
HA(g)	**-**	0.1	**-**	0.05
CS(g)	**-**	**-**	0.1	0.05
NMP(mL)	10	10	10	10

### Morphology characterization of microspheres

The microspheres were visualized and photographed using optical microscope (Mengou, China). The diameter of the microspheres was measured by grain size shape instrument R-2000 (M.I.P TECHNOLOGY [Changzhou] CO, LTD). The resultant microspheres were put into the liquid nitrogen to quick-freeze, and cut the microspheres by sharp blade to display the cross section of microspheres. Then, put the frozen microspheres to the freezing dryer (FD-1D-50, Beijing BYK Co., Ltd) for 3 h. Using field emission scanning electron microscopy (SEM, Hitachi S3000 N) to observe the surface and cross section of the microspheres with platinum spraying on the microspheres. EDS mapping was performed at 15 kV.

### Characterization of hydrophilicity and mechanical properties

To evaluate the hydrophilicity of the materials, suspensions of PGCL, HA/PGCL, CS/PGCL and HA/CS/PGCL were uniformly coated onto siliconized glass slides and immersed in a 50% ethanol solution to form films. After drying, the films were used for subsequent hydrophilicity measurements. Hydrophilicity of the material was measured using a DSA100 (Kruss, Germany) at room temperature. The surface mechanical properties of the materials were evaluated using a TriboIndenter TI Premier (Bruker). The elastic modulus and hardness of the material surfaces were recorded.

### Degradation characteristics of microspheres

Degradation experiments were designed according to the Chinese medical industry standard “YY/T 1806.1-2021.” The PGCL, HA/PGCL, CS/PGCL and HA/CS/PGCL materials were sterilized using ultraviolet radiation and 75% ethanol, then, placed in a vacuum drying oven at room temperature for 8 h for standby. Phosphate buffered saline (PBS, pH 7.4 ± 0.05) was used as the degradation medium. Samples were immersed in 50 mL centrifuge tubes at a ratio of 6 mg material to 1 mL PBS solution, sealed and incubated in a 37°C constant temperature incubator. The entire degradation medium was replaced every 14 days. Solution pH changes were monitored using a pH meter at weeks 2, 4, 6, 8, 10 and 12. Three parallel samples were taken for each group at each time point. At weeks 4, 8 and 12, samples were collected, rinsed three times with deionized water, freeze-dried to constant weight and mass loss rates calculated using the formula as follow:


Mass Loss Rate (%)=[(Initial Mass—Residual Mass)/Initial Mass]×100%.

Degradation morphologies were observed using scanning electron microscopy (SEM). The inductively coupled plasma resonance (ICP; ICP-MS, Thermo Scientific Xseries II) was used to accurately measure the Ca content in degradation solution to observe the relese of Ca^2+^.

### *In vitro* mineralization characteristics of microspheres

The *in vitro* mineralization experiment was performed in accordance with the Chinese group standard T/CSBM 0026-2022. Firstly, the PGCL, HA/PGCL, CS/PGCL and HA/CS/PGCL samples were sterilized by immersion in 75% ethanol for 30 min, then, dried in a vacuum drying oven at 60°C for 24 h for later use. The mineralization solution was SBF (pH 7.40 ± 0.05), with a Ca^2+^ concentration of 2.5 mmol/L, a PO_4_³^-^ concentration of 1.0 mmol/L and other ion compositions consistent with human serum. 0.1 g of each dried powder was placed in 50 mL centrifuge tubes and freshly prepared mineralization solution was added in a ratio of 100 mL of solution per 1 g of material. The tubes were sealed and placed in a constant temperature shaker at 37°C, where they were shaken continuously at 120 rpm for 7 and 14 days. During the culture period, 50% of the volume of fresh mineralization solution was added every 24 h to maintain stable ion concentrations. At the end of the culture period, the samples were collected by centrifugation, washed repeatedly three times with deionized water, freeze-dried for 48 h, and then, weighed to observe any mass changes. A scanning electron microscope (SEM, with an accelerating voltage of 15 kV) was used to observe the morphology of the mineralized products on the surface of the samples, to check for the formation of hydroxyapatite crystals, and to characterize them using EDS. Prior to testing, the samples were sputter-coated with gold, and five random fields of view were selected for each sample for observation and recording.

### Cell culture and cytocompatibility evaluation

MC3T3-E1 pre-osteoblasts were cultured in DMEM (Gibco) containing 10% fetal bovine serum and 1% penicillin-streptomycin at 37°C under 5% CO_2_. For microsphere-based cultures, sterilized microspheres (immersed in 75% ethanol for 2 h under UV followed by PBS washing) were placed in 48-well plates and seeded with 1 × 10^4^ cells/well in 500 μL medium. Cell proliferation was measured at Day 1, 3 and 7 using the CCK-8 assay, where absorbance at 450 nm was recorded after 2 h incubation with 10 μL CCK-8 reagent. Live/dead staining was performed by incubating cells with 2 μM calcein-AM and 3.2 μM propidium iodide in PBS for 15 min at 37°C, followed by fluorescent imaging under a Nikon TE-2000U microscope to visualize live (green) and dead (red) cells.

### ALP staining and activity

The MC3T3-E1 cells at a density of 5 × 10^4^ cells per well (*n* = 3) were evenly mixed with the samples and seeded in the 48-well plate using DMEM cell culture medium. After incubation for 7 and 14 days, alkaline phosphatase (ALP) staining was evaluated by using kits purchased from Beyotime (Shanghai, China). The procedure of ALP staining experiment is as follows. Samples were fixed with 4% paraformaldehyde for 20 min at room temperature, rinsed three times with PBS, and stained using an NBT/BCIP chromogenic kit according to the manufacturer’s protocol. Microspheres were incubated with the chromogenic solution in the dark at room temperature until color development stabilized, and images were captured under an optical microscope (Mengou, China). For ALP quantification, cells were lysed with RIPA buffer after three PBS washes. Cell lysates underwent three freeze-thaw cycles (−80°C/20°C), followed by centrifugation at 1000 × g for 10 min to collect supernatants. Supernatants were mixed with p-nitrophenyl phosphate (pNPP) substrate for ALP activity detection and bicinchoninic acid (BCA) solution f or total protein quantification. After 30 min incubation at 37°C, absorbance was measured at 405 nm (pNPP) and 562 nm (BCA) using a microplate reader. Relative ALP activity was calculated as the ratio of OD405/OD562.

### Quantitative real-time PCR

MC3T3-E1 cells were seeded in 6-well plates at 2 × 10^5^ cells with 2 mL medium per well. Osteogenic differentiation potential of microspheres was evaluated by analyzing runt-related transcription factor 2 (Runx2), osteopontin (OPN), osteocalcin (OCN) and collagen-I (Col-I) expression via real-time PCR at 14 and 21 days. Cells were detached from materials using trypsin, followed by RNA extraction with TRIzol reagent according to the manufacturer’s protocol. Briefly, the collected cells were transferred to a 1.5 mL enzyme-free centrifuge tube and washed thrice with PBS. Then, cells were lysed in 1 mL TRIzol, with complete lysis achieved via two cycles of freezing at −80°C and thawing at 37°C. Next, chloroform (200 μL) was added to the lysate, vortexed vigorously for 15 s and incubated at room temperature for 5 min to induce phase separation. After centrifugation at 4°C and 12 000 g for 15 min, the upper aqueous phase (containing RNA) was transferred to a new RNase-free tube, mixed with 500 μL isopropanol by inversion. The mixture was allowed to stand at room temperature for 10 min to precipitate RNA. Following centrifugation at 4°C, 12 000 × g for 10 min, the RNA pellet was washed with 75% RNase-free ethanol to remove residuals. Finally, after centrifugation at 4°C, 7500 × g for 5 min, ethanol was discarded, residual ethanol was removed via brief centrifugation in a laminar flow hood, and the RNA pellet was air-dried for subsequent use. RNA quality and concentration were assessed using a NanoDrop spectrophotometer (Infinite M200, Tecan). Reverse transcription was performed on RNA samples using the PrimeScript RT Reagent Kit with gDNA Eraser on a thermal cycler. Real-time PCR was conducted with the Mx3005P system (Agilent) using SYBR Premix Ex Taq RR420A, following a protocol of 95°C initial denaturation (30 s), 40 cycles of 95°C (5 s) and 56°C (34 s), followed by melt curve analysis (95°C/15 s, 56°C/60 s, 95°C/15 s). Gene-specific primers for glyceraldehyde-3-phosphate dehydrogenase (GAPDH) and target genes were designed by Comate Bioscience Co., Ltd (Table 2).

**Table 2:** Sequences of primers for the qRT-PCR.

Gene	Forward primer sequence	Reverse primer sequence
RUNX2	5-GCCCTCATCCTTCACTCCAAG-3′t	5-GGTCAGTCAGTGCCTTTCCTC-3′t
OPN	5-TCAGGACAACAACGGAAAGGG-3′t	5-GGAACTTGCTTGACTATCGATCAC-3′
OCN	5-AAGCAGGAGGGCAATAAGGT-3′t	5-TTTGTAGGCGGTCTTCAAGC-3′t
Col-1	5-CGCTGGCAAGAATGGCGATC-3′t	5-ATGCCTCTGTCACCTTGTTCG-3′t
GAPDH	5-CAACCTGGTCCTCAGTGTAGC-3′C	5-CGTGCCGCCTGGAGAAACCTGCC-3′3

All data were normalized to GAPDH expression. The gene expression levels were obtained using the threshold cycles (Ct). Relative transcript quantities were calculated by the ΔΔCt method.

### *In vivo* evaluations based on rabbit orbital bone defect repair model

Animal experiments have been approved by the ethical committee of Changchun Institute of Applied Chemistry Chinese Academy of Sciences and confirm compliance with all relevant ethical regulations (20240086). According to the before rabbit orbital bone defect repair model [1, 44], a total of 10 male New Zealand rabbits weighing 2.5–3.0 kg (20 orbital bones) were randomly assigned to five groups: blank control, PGCL, HA/PGCL, CS/PGCL and HA/CS/PGCL. Before surgery, rabbits were anesthetized, and 5-mm diameter bone defects were created on bilateral orbital margins using a dental drill with continuous sterile saline irrigation for cooling. PGCL, HA/PGCL, CS/PGCL and HA/CS/PGCL were implanted into respective defects. After microsphere implantation during the surgical procedure, the endothelial layer of the rabbit’s orbit was first sutured to secure the position of the microspheres, followed by suturing the outer layer. Postoperatively, antibiotics (gentamicin 5 mg/kg intramuscularly, once daily for 3 days) and tobramycin eye drops (three times daily for 7 days) were administered to prevent infection. Daily observations included wound inflammation, healing, infection, implant rejection and morphological changes. Small animal *in vivo* Micro-CT was used to observe the healing of the surgical site biweekly. Micro-CT (Perkinelmer, Japan) was used to evaluate new bone formation within the defect. The reconstructed data were analyzed using CT-VOX and CT-VOL programs. Quantitative parameters included the ratio of new bone volume to total volume (BV/TV), trabecular thickness (Tb.Th), trabecular number (Tb.N) and trabecular separation (Tb.Sp). At week 10, Orbital tissues were exposed by incising periorbital skin along the eyelid, separating subcutaneous tissues and examining peri-implant tissue reactions. Implants with surrounding tissues were harvested, fixed in 4% formaldehyde for 24 h, dehydrated, cleared and paraffin-embedded. Serial 7 μm sections were stained with H&E, Masson’s trichrome and Sirius red, then scanned using panoramic microscopy (Tissue Gnostics, Austria) to assess repair site growth.

### Statistical analysis

The data were analyzed using Origin 2019 and are presented as the mean ± standard deviation. The statistic difference was evaluated by variance analysis (ANOVA one-way, Origin 2019). A value of P* < 0.05 was regarded as statistically significant.

## Results and discussion

### Characterization of spherical CS mesocrystals

**Figure 1:**
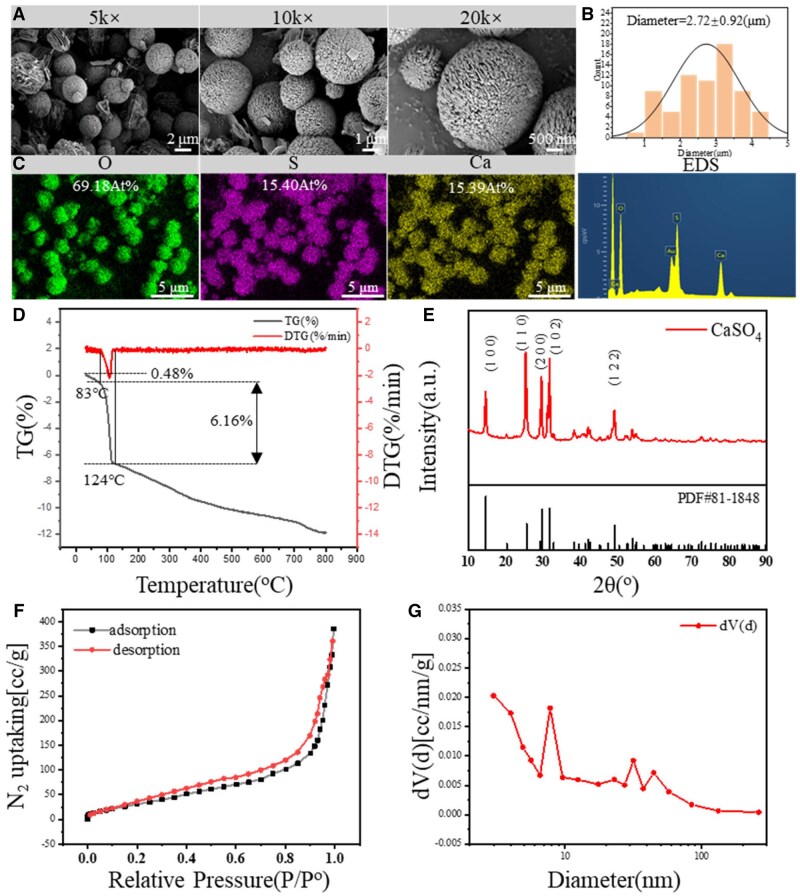
(**A**) Spherical CaSO_4_ mesocrystal at different magnifications and all the scale bar lengths are 2 μm, 1 μm and 500 nm, respectively. (**B**) The statistical size distribution of spherical CaSO_4_ mesocrystals. *N* = 25. (**C**) Mapping of spherical CaSO_4_ mesocrystal. (**D**) The TG of spherical CaSO_4_ mesocrystal. (**E**) The XRD of spherical CaSO_4_ mesocrystal. (**F**) The nitrogen isothermal adsorption desorption curve of spherical CaSO_4_ mesocrystal. (**G**) Pore distribution on the surface of spherical CaSO_4_ mesocrystal.

Figure 1A demonstrates that the prepared microspheres exhibit excellent sphericity. Observation at 20 000× magnification reveals numerous grooves on the surface of spherical CS mesocrystals, which are composed of tiny structural units, consistent with mesocrystalline characteristics. Figure 1B presents the particle size distribution of spherical CS mesocrystals. The average particle size is 2.72 μm. Spherical CS mesocrystals form via non-classical particle-mediated crystallization and may cease growth after reaching a certain size, leading to size concentration in the range of 3.0–3.6 μm. Figure 1C shows the element mapping and EDS spectra of spherical CS mesocrystals. The main elemental compositions are O (69.19 At%), S (15.40 At%) and Ca (15.39 At%) and atomic ratio of O: S: Ca is 4.5:1:1, which match the theoretical values of calcium sulfate hemihydrate.

Figure 1D depicts the thermogravimetric analysis (TGA) curve of spherical CS mesocrystals. A weight loss of 0.48% occurs at 20–81°C, primarily due to the decomposition of adsorbed water on the whisker surface. A distinct weight loss of 6.16% is observed at 83–124°C, which highly coincides with the theoretical crystal water content (6.2%) of calcium sulfate hemihydrate, confirming the presence of crystal water in spherical CS mesocrystals and indicating that the product is calcium sulfate hemihydrate (CaSO_4_·0.5H_2_O). The XRD results in Figure 1E reveal that the diffraction peak positions of the prepared calcium sulfate microspheres are consistent with those in the standard pattern of hemihydrate gypsum. However, compared with the standard card, the peak intensity corresponding to the (1 0 0) crystal plane is decreased, while that of the (1 1 0) crystal plane is increased. This indicates that the growth of the (1 0 0) crystal plane of hemihydrate calcium sulfate in the spherical CS mesocrystals is inhibited, whereas the growth of the (1 1 0) crystal plane is enhanced, which is consistent with the arrangement result of the nanorods shown in Figure 1A.

Figure 1F displays the nitrogen adsorption-desorption isotherm of calcium sulfate spherical CS mesocrystals. The curve shows a downward convex shape in the low-pressure region and directly forms a hysteresis loop at relative pressures of 0.2–0.9. According to IUPAC classification, this curve belongs to type V isotherm, indicating weak interactions between nitrogen and mesoporous surfaces. The specific surface area of spherical CS mesocrystals is calculated as 106.772 m^2^/g. Figure 1G presents the pore size distribution results of the spherical CS mesocrystals, showing that the pore size range is mainly between 2 nm and 10 nm, with an average pore size of 1.87 nm. These porous structures may lead to a faster degradation efficiency of spherical CS mesocrystals, making them more suitable for calcium ion release.

### Hydrophilicity and mechanical properties of composite materials

**Figure 2:**
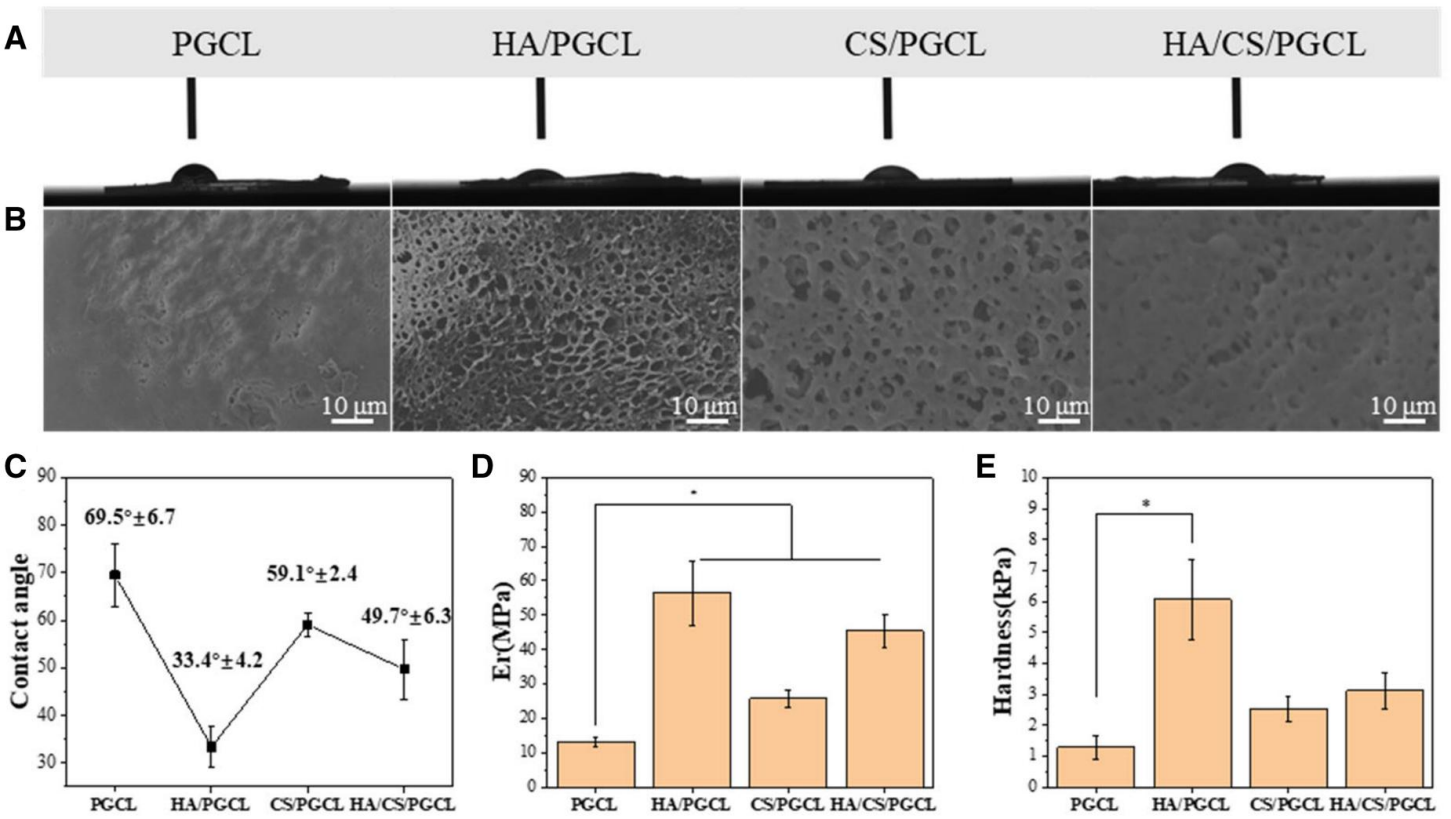
(**A**) Images of water droplets spreading on different substrates. (**B**) SEM of film surface. (**C**) Statistical results of contact angles on different substrate materials. (**D**) Results of the elastic modulus on different microsphere surfaces. (**E**) Results of the hardness on different microsphere surfaces. *P < 0.05.

**Figure 3:**
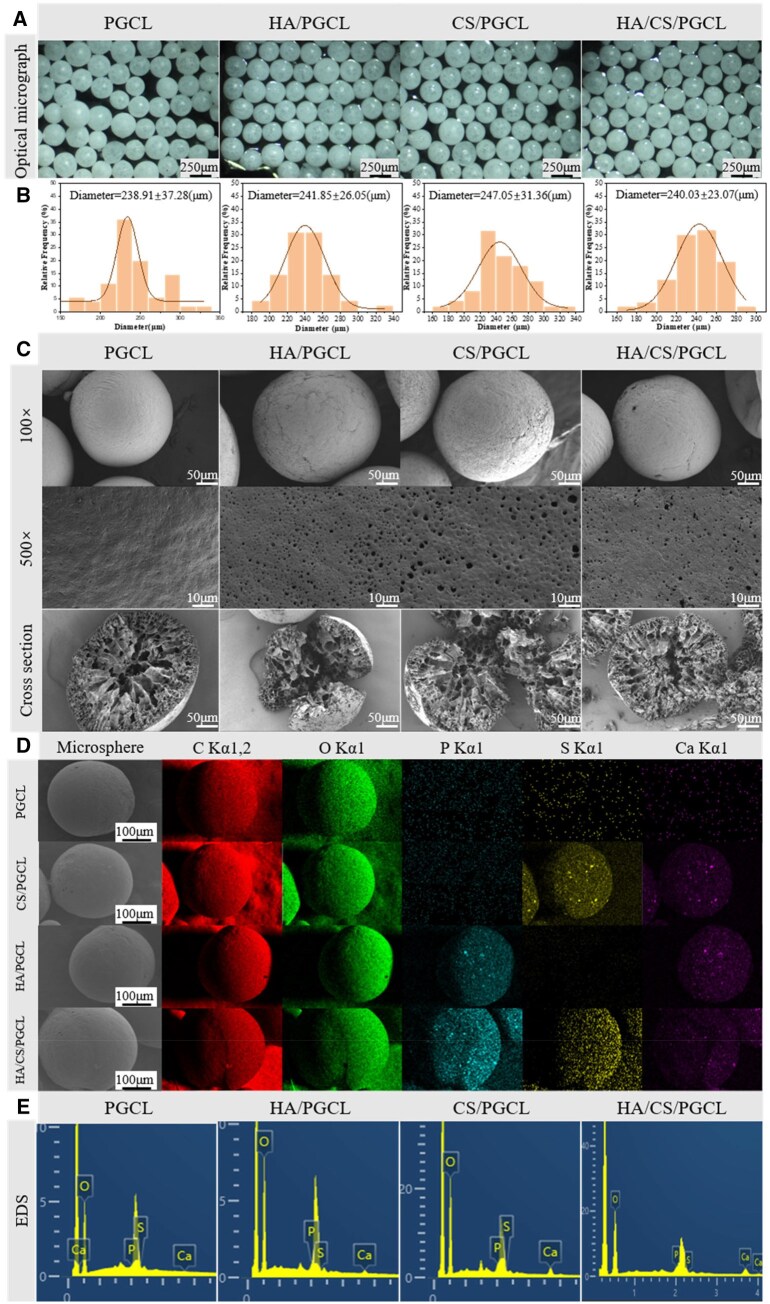
(**A**) Micrographs of different microspheres. The bars of the micrographs are 250 μm. (**B**) The statistical size distribution of microspheres. *N* = 25. (**C**) Morphology and cross section of microspheres, the bars are 50 μm. (**D**) Mapping of microspheres and (**E**) EDS of microspheres.

Figure 2A and C display the wettability images and contact angle data of composite materials. And Figure 2B shows the surface structure of films produced as described in Characterization of hydrophilicity and mechanical properties. As it shows that the structure of the film surface owns the same structure as the surface of the microspheres. Only a small amount of nanoscale pore structures are observed on the surface of microspheres in the PGCL group, while microspheres in the HA/PGCL, CS/PGCL and HA/CS/PGCL groups all exhibit distinct distributions of relatively large pores. The surface structural characteristics of microspheres across all groups show high consistency, and the chemical composition of the prepared films is identical to that of the microspheres. Based on these facts, it can be concluded that the hydrophilicity/hydrophobicity of the film surface is representative of that of the microsphere surface in this study. A 2 μL deionized water droplet was deposited on the polymer surface at 25°C. The droplet exhibited a clear and axisymmetric profile, with no visible contamination on the sample surface. The PGCL group showed a higher droplets curvature, with a contact angle of 69.5° as measured by software analysis. PGCL is a hydrophobic polyester material, while the sample shows some hydrophilicity, which may be related to the increased roughness caused by the material’s porosity [45]. In contrast, the HA/PGCL group exhibited reduced droplet curvature and greater spreading, and had a contact angle of 33.4°, indicating enhanced hydrophilicity. The CS/PGCL group had intermediate droplet curvature, with a contact angle of 59.1°, while the HA/CS/PGCL group showed the lowest contact angle of 49.7°, reflecting a further improvement in hydrophilicity. As shown, incorporating HA and calcium sulfate significantly reduces the contact angle, enhancing the material’s hydrophilicity, with the HA/PGCL group exhibiting greater hydrophilicity than the CS/PGCL group. The EDS image in Figure 3D shows HA and CS exposure on the microsphere surface. The hydrophilicity of HA and CS further enhances the material surface hydrophilicity [46, 47]. The increased hydrophilicity is beneficial for *in vitro* mineralization and cell adhesion [48], thus, increasing the material’s bioactivity. Figure 2C and D present the elastic modulus and hardness of the microsphere surfaces. According to the literature [49, 50], HA and CS can enhance the mechanical properties of materials. The results indicate that adding HA and calcium sulfate increases both the surface elastic modulus and hardness, and the results of this experiment are consistent with the literature. As indicated by the above results, HA and calcium sulfate enhance the material’s surface hydrophilicity and mechanical properties, thereby improving the cellular microenvironment on the material’s surface.

### Morphology of microspheres

Figure 3A displays photomicrographs of the microspheres taken under an optical microscope. It is evident from the figure that the prepared microspheres exhibit good sphericity. Figure 3B is the result of the size distribution of microspheres. The narrow range of microsphere size distribution indicates a uniform size among the microspheres. The diameters of the microspheres are approximately 240 µm. Figure 3C presents the surface morphology and cross-sectional SEM images of the microspheres. PGCL microspheres exhibit a smooth surface, while HA/PGCL, CS/PGCL and HA/CS/PGCL microspheres show slightly rough surfaces, and at a magnification of 500×, min pores can be observed on the surface of the microspheres. Moreover, the pore sizes on the surfaces of HA/PGCL, CS/PGCL and HA/CS/PGCL microspheres are significantly larger than those on the surfaces of PGCL microspheres, likely due to the incorporation of HA and CaSO_4_ mesocrystalline microspheres affecting the curing of the PGCL/NMP solution. Cross-sectional observations reveal a pore gradient: larger internal pores and smaller surface-near pores. As reported in the literature [51], when a polymer solution comes into contact with water or body fluids, the solvent (e.g. NMP) diffuses into the surrounding environment, while water or body fluids permeate the polymer solution, creating a thermodynamically unstable state. This instability triggers phase separation, leading to the formation of polymer-rich and polymer-lean phases. During this process, droplets of the polymer-lean phase form within the polymer-rich phase and gradually grow. As the solvent continues to diffuse and water permeates further, these droplets solidify over time, resulting in the formation of pores. From the Figure 3D and E, in the S and Ca mapping of the PGCL group, visible microsphere outlines are software-generated pseudocolors. EDS of the same region (Figure 3E) shows no characteristic S or Ca peaks, confirming these elements are absent in PGCL microspheres. In contrast, HA/PGCL group’s elemental mapping exhibits distinct brighter regions (indicating P and Ca distribution), and CS/PGCL and HA/CS/PGCL groups show the same mapping-EDS consistency. Mappings and EDS confirm the successful incorporation of HA and calcium sulfate nanocrystals, showing P, S and Ca distribution on the microsphere surfaces.

### *In vitro* degradation of microspheres

**Figure 4:**
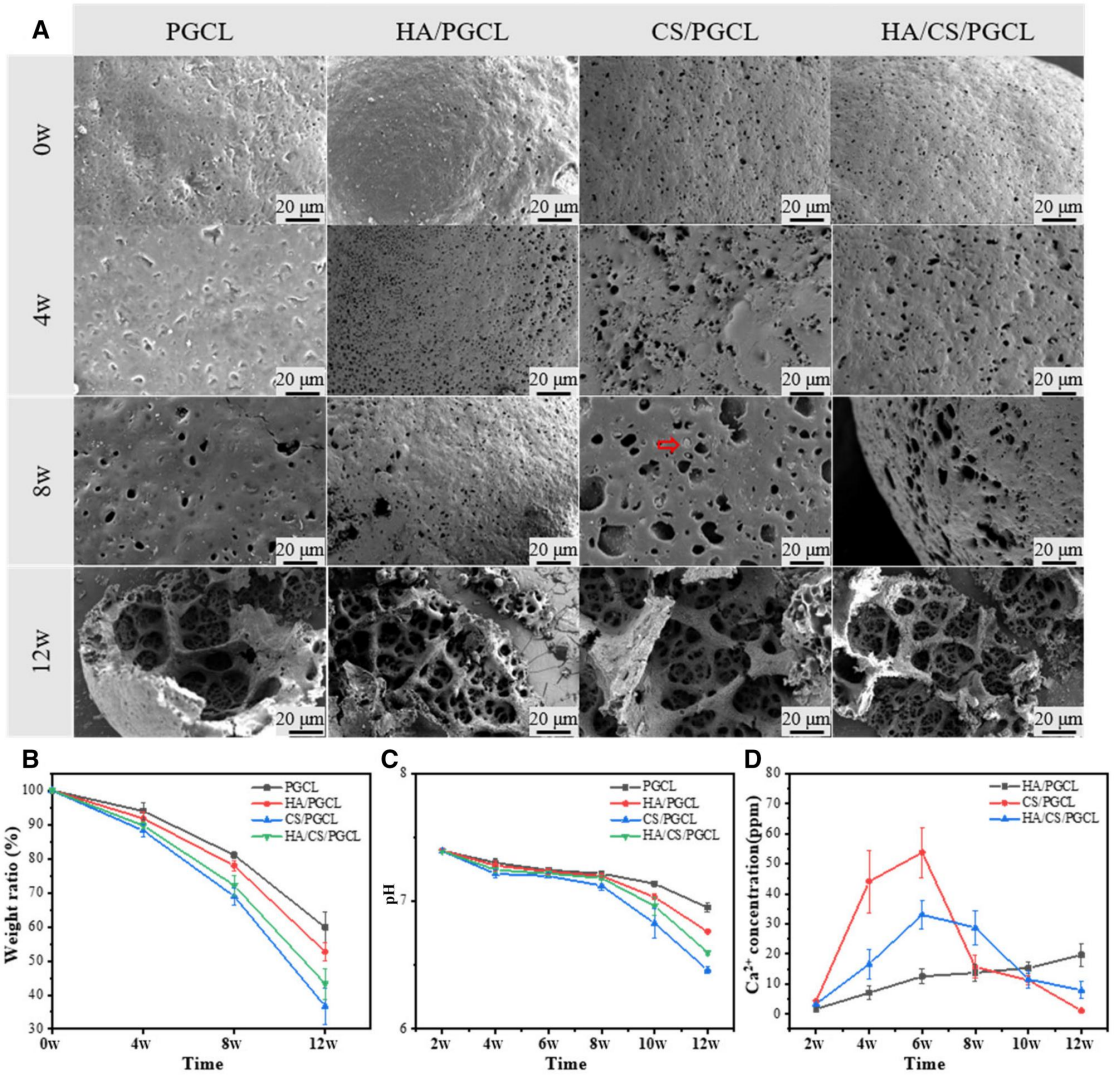
(**A**) Time-sequential degradation images of different microspheres in PBS solution at body temperature (37°C) during 12 weeks, the scale bar lengths are 20 μm. (**B**) Weight variation of different microspheres during a 12-week degradation period. (**C**) Changes in pH of PBS of different microspheres during a 12-week degradation period. (**D**) Ca^2+^ release curve of HA/PGCL, CS/PGCL and HA/CS/PGCL microspheres during a 12-week degradation period.

PGCL undergoes hydrolysis via cleavage of its easily hydrolyzable ester bonds (–COO–), forming fatty alcohols (–OH) and carboxylic acids (–COOH), as previously reported [52]. Figure 4A shows the degradation behaviors of PGCL, HA/PGCL, CS/PGCL and HA/CS/PGCL groups immersed in phosphate-buffered saline (PBS, pH 7.4) at 37°C (physiological temperature) at 4, 8 and 12 weeks. At the 4th week, the pores on the material surface began to enlarge, the surface became rougher and very few cracks were generated. At the 8th week, it can be seen that the pores on the microsphere surface further increased, and in the CS/PGCL group, the degradation of calcium sulfate mesocrystal microspheres and the resulting pores can be observed as indicated by the red arrow. This indicates that calcium sulfate mesocrystal microspheres can provide a pore-forming effect after degradation, thus, achieving the purpose of spatial regulation of pore structure. At the 12th week, it can be seen that most of the microspheres had degraded, the spherical shape had been destroyed, and there were a large number of cracks and pores left by degradation on the surface. The HA/PGCL group, CS/PGCL group and HA/CS/PGCL group had a deeper degree of fragmentation compared to the PGCL group.

Figure 4B and C show mass loss and pH changes during degradation, respectively. In Figure 4B, mass loss and its rate increased over time, likely due to material degradation and increased specific surface area. During *in vitro* degradation, solutions were refreshed biweekly, with pH measured at the same intervals (Figure 4C). As degradation produced carboxylic acids (–COOH), solution pH decreased over time. At 8 weeks, pH declined slightly (7.12–7.22), remaining within the buffer’s buffering range. At 10 weeks, PGCL and HA/PGCL groups maintained near-neutral pH (7.13 and 7.03, respectively), while CS/PGCL and HA/CS/PGCL groups became acidic (6.82 and 6.96), indicating accelerated degradation and accumulated acidic byproducts. By 12 weeks, all groups showed acidic pH (PGCL: 6.95; HA/PGCL: 6.76; CS/PGCL: 6.46; HA/CS/PGCL: 6.6), reflecting rapid degradation and substantial acidic product release-consistent with SEM observations and mass loss data. Figure 4D shows the calcium ion release curve, with measurements taken biweekly. HA/PGCL showed a steady increase in calcium ion release, while CS/PGCL and HA/CS/PGCL exhibited an initial rise followed by a decline. Between 4 and 8 weeks, CS/PGCL and HA/CS/PGCL released more calcium ions than HA/PGCL: PGCL degradation exposed CS (faster degradation than HA), enabling earlier calcium ion release. After 8 weeks, CS/PGCL calcium ion release decreased as most CS degraded, whereas HA/CS/PGCL maintained higher levels due to sustained HA degradation. Overall, HA and CS modification regulated PGCL degradation to maintain high calcium ion release over 12 weeks, with HA/CS/PGCL demonstrating sustained calcium ion supply capacity for bone repair.

In summary, through an in-depth analysis of the multi-group data in Figure 4, the degradation characteristics of PGCL and its composite materials can be obtained. From the evolution of the material surface microstructure, it can be seen that as time progresses, the materials in all experimental groups show a gradual deterioration trend. The HA/PGCL, CS/PGCL and HA/CS/PGCL groups exhibit a deeper degree of fragmentation compared to the PGCL group, which suggests that the additives hydroxyapatite (HA) and calcium sulfate mesocrystal microspheres (CS) significantly affect the structural integrity of the materials and accelerate their decomposition process. The mass loss data intuitively reflect that material degradation intensifies over time and is closely related to the increase in specific surface area. Notably, the CS/PGCL group shows the most pronounced changes compared to other groups, which is attributed to the pores generated by the degradation of calcium sulfate mesocrystal microspheres increasing the material’s specific surface area, corroborating the structural changes observed. The monitoring results of the degradation solution pH serve as critical evidence: through correlation analysis with other data, they precisely identify differences in the degradation rates of different materials at different stages. For example, at weeks 10 and 12, the rapid pH decrease in the CS/PGCL group corresponds to severe surface damage and substantial mass loss of the material.

### *In vitro* mineralization of microspheres

**Figure 5:**
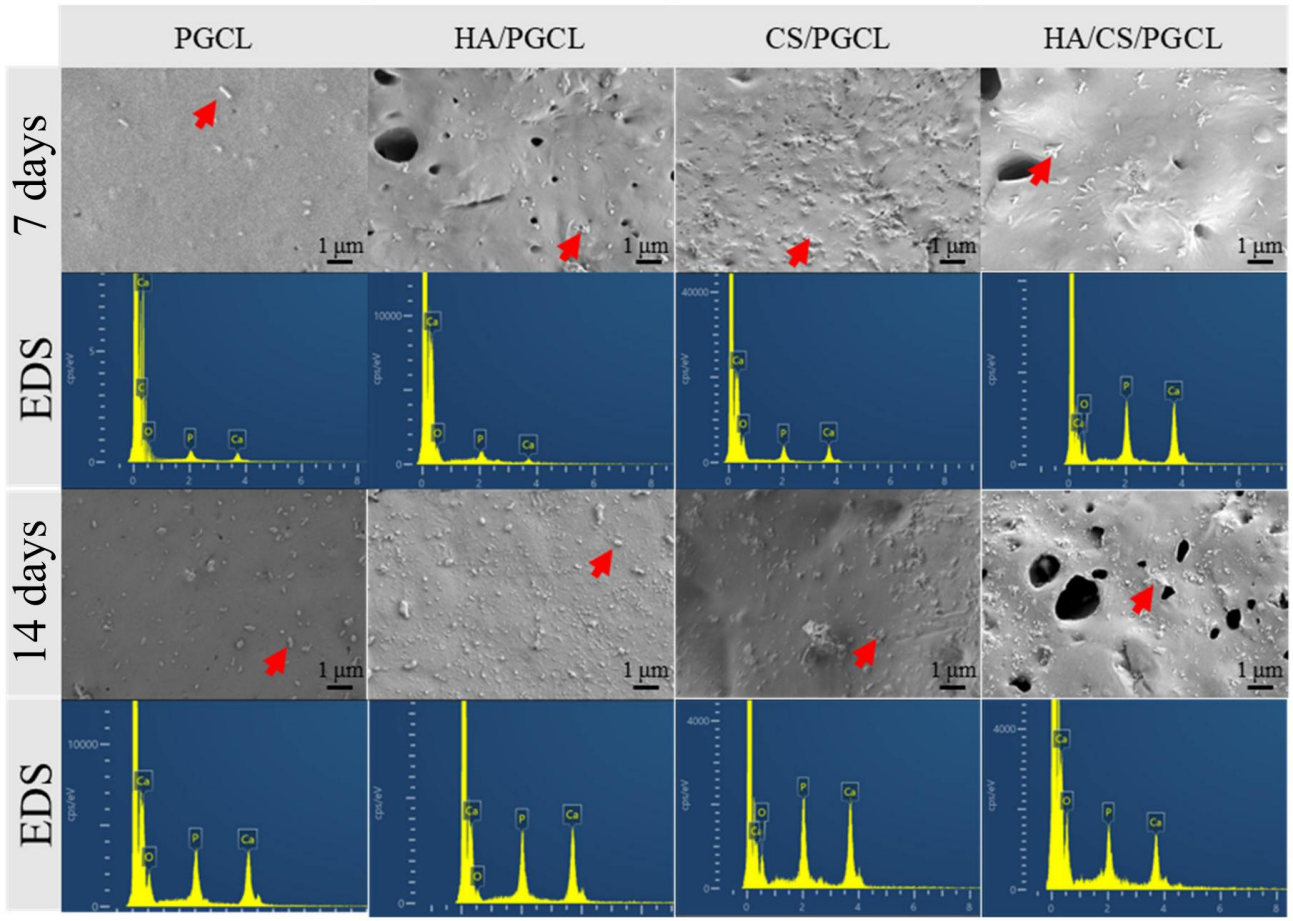
SEM images and corresponding EDS mappings of the microsphere surface after *in vitro* mineralization at 7 and 14 days, the scale bar lengths are 1 μm.

*In vitro* mineralization is one of the commonly used characterizations to evaluate the osteogenic ability of materials. Figure 5 shows the scanning electron microscope (SEM) images of the microspheres’ surfaces after 7 and 14 days of mineralization, and the energy-dispersive X-ray spectroscopy (EDS) characterization was performed on the particles indicated by the red arrows, with the corresponding results as follows. It can be seen from the SEM images that irregular small particles appeared on the material surface on the 7th day. The PGCL group had fewer surface particles, while the HA/PGCL, CS/PGCL and HA/CS/PGCL groups had more particles on their surfaces than the PGCL group. Moreover, EDS analysis was carried out on the particles, and the main elemental composition of the particles was P, Ca and O. The calcium-phosphorus ratio (Ca/P ratio) data are detailed in Table 3. On the 7th day of culture, the Ca/P ratios of all experimental groups were within the range of 1.59–1.64. It is known that the theoretical Ca/P ratio of crystalline hydroxyapatite (HA) is 1.67. The slight deviation between the measured values in this study and this theoretical value is presumably attributed to the amorphous phase formed in the early stage of mineralization. On the 14th day, there were still few mineralized particles formed on the surface of the PGCL group, indicating that the PGCL material had insufficient mineralization ability. In the HA/PGCL, CS/PGCL and HA/CS/PGCL groups, however, more mineralized particles were formed on the surface. The EDS analysis results showed that the energy spectra of the particles were more consistent with HA. Additionally, the Ca/P ratios of the HA/PGCL and HA/CS/PGCL groups were both 1.67, and that of the CS/PGCL group was 1.66, suggesting that the surface particles were relatively close to crystalline HA. By detecting the mass changes of the materials after 7 and 14 days and the result was shown in Table 4, it was found that the masses of PGCL and HA/PGCL increased slightly, while the masses of CS/PGCL and HA/CS/PGCL groups decreased slightly, which did not conform to the mass change law of mineralization. This may be related to the disintegration of CS. It can be seen from the above results that the PGCL material itself basically does not possess the *in vitro* mineralization ability. However, there are still a small number of mineralized particles deposited on its surface, which may be related to the increased hydrophilicity of the material. The above results suggest the addition of HA and CS can enhance the *in vitro* mineralization ability of the material, demonstrating its potential for a bone repair material.

**Table 3:** Ca/P of the EDS of microspheres.

	PGCL	HA/PGCL	CS/PGCL	HA/CS/PGCL
7 days	1.59 ± 0.011	1.64 ± 0.006	1.63 ± 0.004	1.63 ± 0.002
14 days	1.65 ± 0.009	1.67 ± 0.003	1.67 ± 0.002	1.67 ± 0.004

**Table 4:** Changes in weight of different microspheres during *in vitro* mineralization at 7 and 14 days.

	PGCL	HA/PGCL	CS/PGCL	HA/CS/PGCL
7 days (%)	101.09 ± 7.69	105.16 ± 2.96	99.16 ± 3.32	100.84 ± 9.95
14 days (%)	103.82 ± 6.54	108.95 ± 2.73	97.36 ± 4.24	99.03 ± 1.32

### Cell proliferation and immunofluorescence cell-staining results

**Figure 6:**
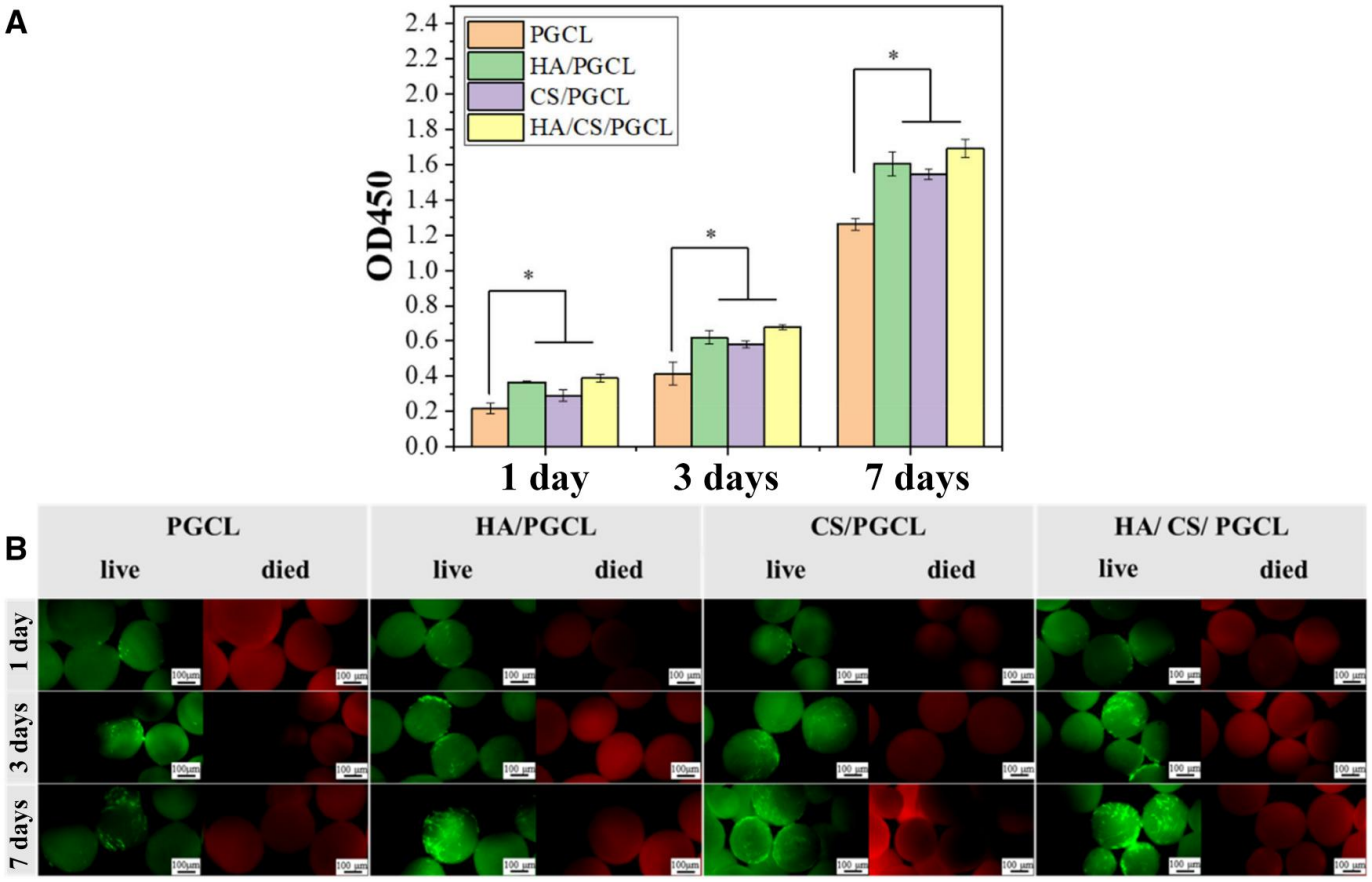
(**A**) CCK-8 test of the proliferation of MC3T3-E1 on the different microspheres for 1, 3 and 7 days; *n* = 3. *P < 0.05. (**B**) Micrographs of the adhesion and growth of MC3T3-E1 on the different microspheres at 1, 3 and 7 days stained with Calcein-AM/PI. All scale bars lengths are 100 μm.

In this study, systematic evaluation of cellular behaviors on microspheres with different material surfaces was conducted through Calcein-AM/PI staining and CCK-8 assays to investigate the influence of material characteristics on cellular viability. As depicted in Figure 6A, CCK-8 assay data corroborated the staining observations. On Day 1, higher OD values in HA/PGCL, CS/PGCL and HA/CS/PGCL groups indicated superior metabolic activity and faster proliferation rates in composite groups. The disparity in OD values between composite groups and PGCL further varied during extended culture. Figure 6B shows the micrographs of the adhesion and growth. Although live/dead fluorescence staining images cannot display the adhesion details of cells on the microsphere surface with the same level of precision as scanning electron microscopy (SEM) images, they nevertheless enable clear visualization of cell spreading and growth status. As Figure 6B depicted, during the initial adhesion phase (Day 1), microscopic examination revealed sparse cell attachment on PGCL microspheres, whereas HA/PGCL, CS/PGCL and HA/CS/PGCL groups exhibited significantly enhanced cellular adhesion. This notable disparity can be attributed to modifications in material surface properties. The incorporation of HA and CS improved surface hydrophilicity, which facilitates adsorption of cell adhesion-related proteins and thereby promotes initial cellular attachment. Moreover, increased surface roughness provided additional anchorage sites for cell spreading. PI staining results demonstrated minimal red fluorescence across all groups, strongly confirming the favorable cytocompatibility of all fabricated materials without inducing cytotoxicity. Upon extending the culture period to Day 3, sustained cellular proliferation was observed with increased cell numbers on all microsphere surfaces, indicating progressive adaptation to the material microenvironment. By Day 7, HA/PGCL, CS/PGCL and HA/CS/PGCL groups exhibited substantially greater cell density compared to PGCL, highlighting the long-term advantages of HA and CS incorporation. HA, as a crucial inorganic component of bone tissue with PGCL optimizes surface microenvironments for prolonged cellular survival and expansion. Physiological mineralization sites to promote cellular differentiation and proliferation. CS, characterized by excellent biodegradability, supplies sulfate ions and nutrients during degradation to stimulate cellular metabolism. The synergistic combination of these component conclusion, the HA/CS-PGCL composites synergistically improved cellular adhesion, proliferation and cytocompatibility through optimized surface hydrophilicity, roughness and biomimetic microenvironments. These modifications provide critical insights for designing advanced biomaterials in tissue engineering applications.

### The ALP activity evaluation

**Figure 7:**
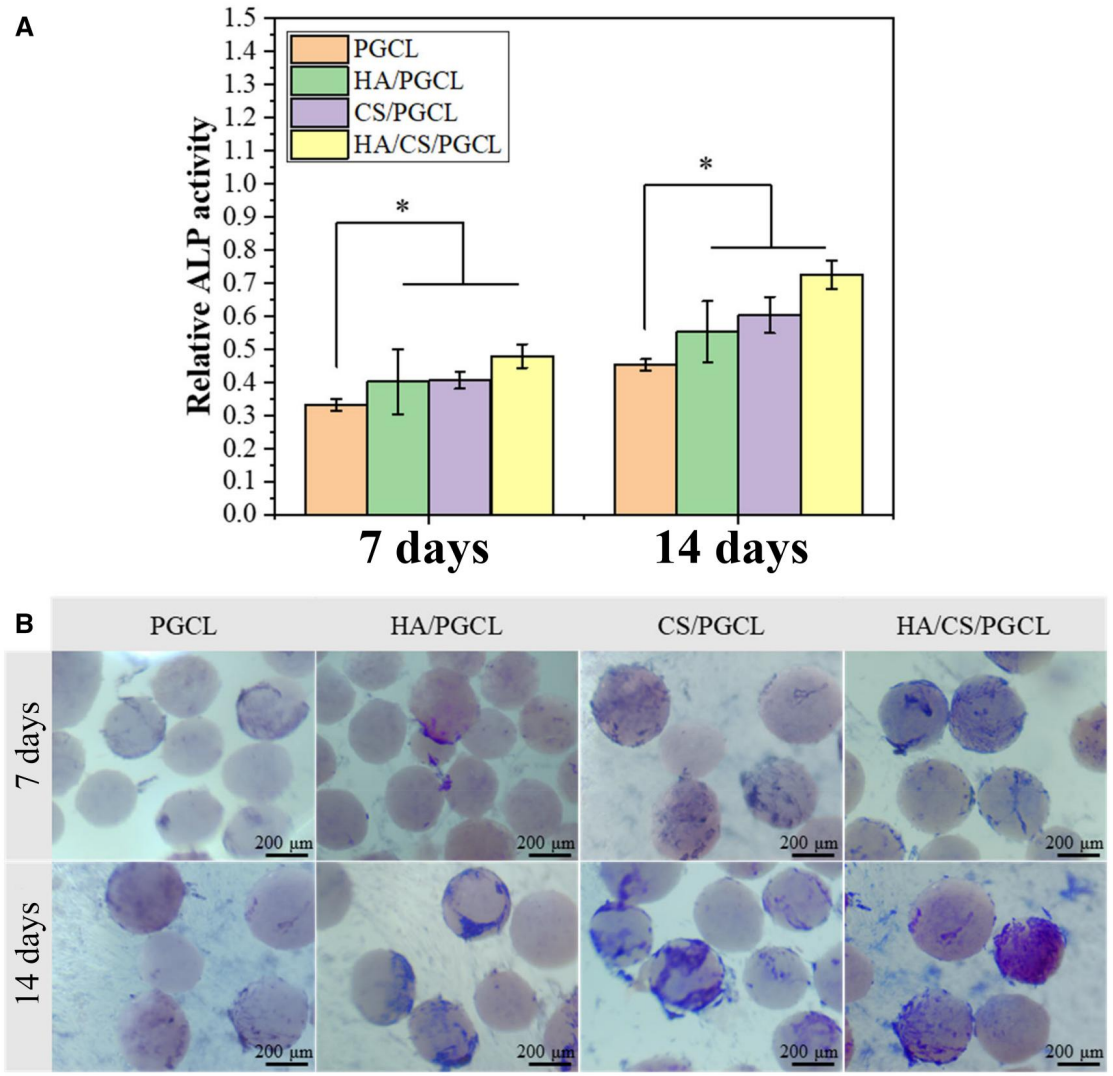
(**A**) The activity of corresponding ALP quantitative evaluation analysis. The scale bar is 200 μm. *P < 0.05. (**B**) The a LP staining of MC3T3-E1 cells cultured on different microspheres for 7 days and 14 days.

Figure 7A shows relatively low ALP activity across all groups at 7 days. However, HA/PGCL, CS/PGCL and HA/CS/PGCL groups exhibited significantly higher activity than pure PGCL (*P* < 0.05). HA/CS/PGCL activity was marginally higher than HA/PGCL and CS/PGCL and no significant difference between the latter two, suggesting HA-CS synergy: HA enhances hydrophilicity and cell affinity for adhesion, while CS-released Ca^2+^ boosts ALP activity. By 14 days, composite groups showed markedly increased ALP activity (*P* < 0.01), with HA/CS/PGCL being highest (∼1.6× pure PGCL). CS/PGCL activity slightly exceeded HA/PGCL. This aligns with osteogenic differentiation kinetics: 7-day focus on adhesion and proliferation while 14-day upregulation of mineralization marker ALP. HA/CS/PGCL superiority may derive from HA providing Ca^2+^/PO_4_³^-^ for mineralization and CS-released Ca^2+^ activating osteoblast calcium signaling.Figure 7B staining correlates with quantitative data. At 7 days, all groups showed faint blue staining (pure PGCL nearly negative), while composite groups had sparse positive regions, indicating limited early osteoblast differentiation. By 14 days, pure PGCL staining increased moderately but weakly. HA/PGCL and CS/PGCL staining expanded and darkened, with HA/CS/PGCL exhibiting dense, deep blue coverage of microsphere surfaces. This confirms HA/CS/PGCL superiority via: (1) HA/CS-released ions activating mineralization enzymes; (2) enhanced roughness/hydrophilicity promoting cell adhesion/spreading [48]; (3) CS-released Ca^2+^ boosting calmodulin activity [53]; (4) improved mechanics activating YAP/TAZ pathways [54].In summary, HA/CS/PGCL synergistically enhances osteoblast ALP activity, showing promise for bone tissue engineering.

### Expressions of osteogenesis-related genes

**Figure 8:**
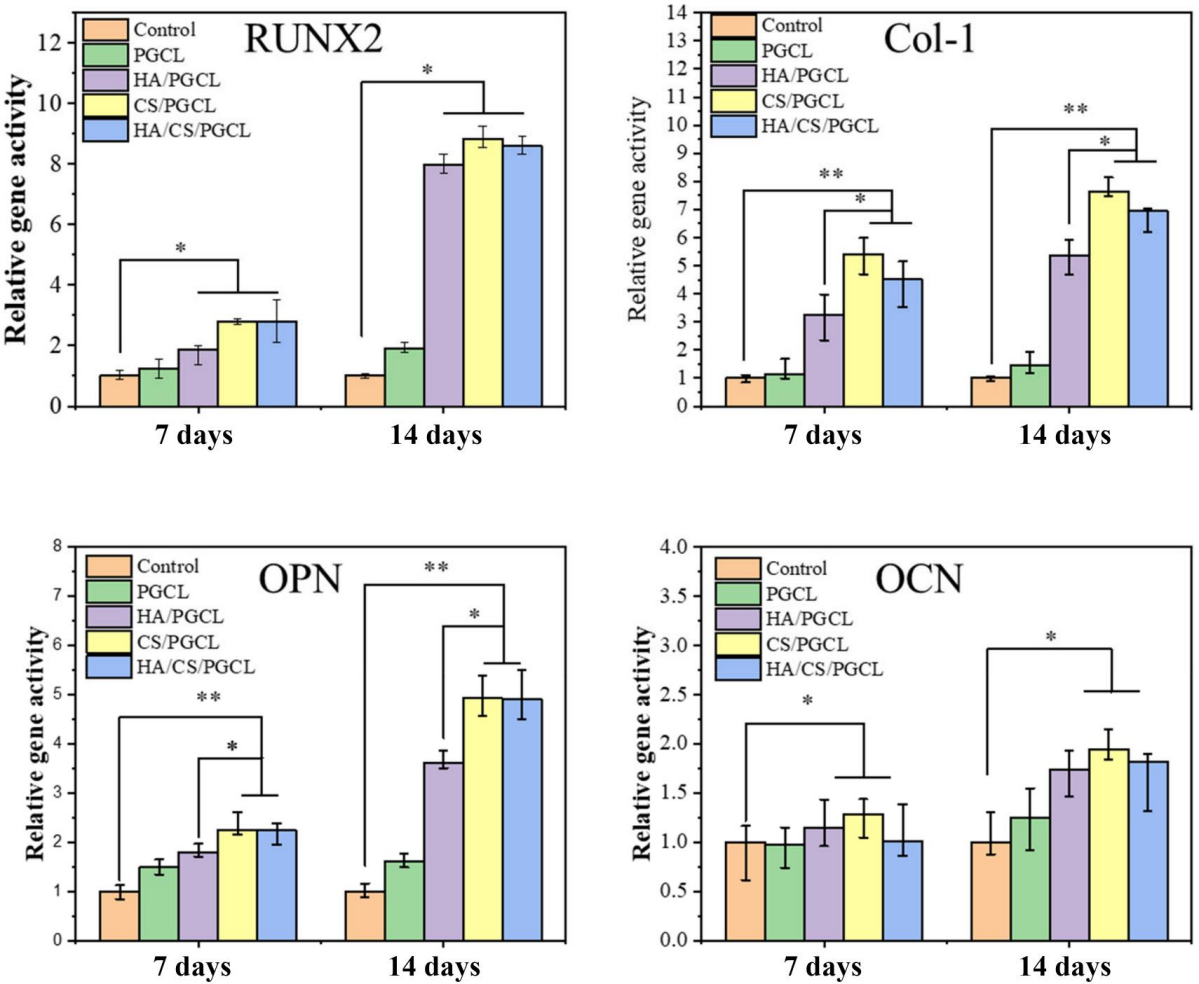
Relative gene expression of RUNX-2, Col-I, OPN and OCN on Day 7 and 14 after seeding. *P < 0.05, **P < 0.01.

To investigate osteogenesis-related gene expression in different material composites and explore their mechanisms in bone formation, osteogenic marker genes (RUNX2, Col-1, OPN, OCN) were monitored, with results shown in Figure 8.

RUNX2, a key osteogenic signaling molecule regulating early bone development, was significantly upregulated in HA/PGCL, CS/PGCL and HA/CS/PGCL groups versus controls at 7 days, with relative activities 1.87-, 2.81- and 2.79-fold higher, respectively. This indicates HA/CS combined with PGCL effectively activates early osteogenic pathways and promotes RUNX2 transcription, likely by providing a favorable microenvironment for cell adhesion/proliferation and initiating osteogenic differentiation. The PGCL group showed only slight upregulation, lacking specific osteogenic signals from HA/CS. By 14 days, RUNX2 expression in composite groups further increased, with relative activities 7.99-, 8.83- and 8.60-fold higher than controls, respectively, reflecting sustained material-induced enhancement of RUNX2 expression via prolonged cell-material interactions, supporting osteoblast maturation and matrix secretion.

Collagen-1 (Col-1), the main organic bone matrix component, reflects osteoblast extracellular matrix synthesis capacity. At 7 days, consistent with RUNX2 trends, HA/PGCL, CS/PGCL and HA/CS/PGCL groups exhibited 3.23-, 5.40- and 4.50-fold higher relative gene activities versus controls; the PGCL group showed a slight increase. This confirms composite materials promote early osteoblast activation and matrix synthesis initiation. CS/PGCL and HA/CS/PGCL groups outperformed HA/PGCL, suggesting CS-derived calcium may provide additional stimuli for Col-1 expression and optimize collagen-related signaling. By 14 days, relative activities were 5.35-, 7.64- and 6.95-fold higher in HA/PGCL, CS/PGCL and HA/CS/PGCL groups, respectively, indicating sustained support for efficient matrix synthesis, with material synergies/differences providing a genetic basis for tailored bone regeneration applications.

Osteopontin (OPN), involved in bone mineralization, cell adhesion and osteoclast regulation, was significantly elevated in composite groups versus PGCL/controls at 7 days, with CS/PGCL and HA/CS/PGCL slightly exceeding HA/PGCL (1.78-, 2.24-, 2.23-fold vs controls). This indicates HA/CS-PGCL enhances early OPN secretion, facilitating cell adhesion and mineralization preparation, likely via CS-released calcium regulating OPN upstream transcription factors. At 14 days, differences widened, with CS/PGCL and HA/CS/PGCL showing comparable expression (3.61-, 4.92-, 4.89-fold vs controls), reflecting mature osteoblast differentiation and sustained OPN expression to regulate cell-matrix/cell-cell interactions, ensuring orderly bone mineralization/remodeling and highlighting composite advantages in long-term osteogenic regulation.

Osteocalcin (OCN), a late osteoblast marker linked to matrix maturation, showed slightly higher expression in CS/PGCL versus other groups at 7 days (no significant differences among others). This suggests CS may prematurely activate late osteoblast maturation, inducing mild early OCN upregulation, while HA effects were not yet evident. By 14 days, OCN significantly increased in HA/PGCL, CS/PGCL and HA/CS/PGCL groups versus PGCL/controls (PGCL showed slight elevation), with relative activities 1.73-, 1.94- and 1.82-fold higher. This indicates composites stimulate late osteoblast maturation, promoting OCN secretion and matrix mineralization, confirming their ability to mimic natural bone formation regulation and providing a molecular basis for optimized bone repair materials.

In conclusion, HA/PGCL, CS/PGCL and HA/CS/PGCL composites exhibited multidimensional regulatory differences and synergies in RUNX2, Col-1, OPN and OCN expression over time. Early RUNX2 activation initiated osteogenic differentiation, followed by Col-1/OPN responses ensuring matrix synthesis and mineralization preparation, with OCN marking maturation completion. CS exerted unique potentiating effects at specific stages, likely via released calcium ions enhancing osteoinduction.

### Evaluation of bone regeneration *in vivo*

**Figure 9:**
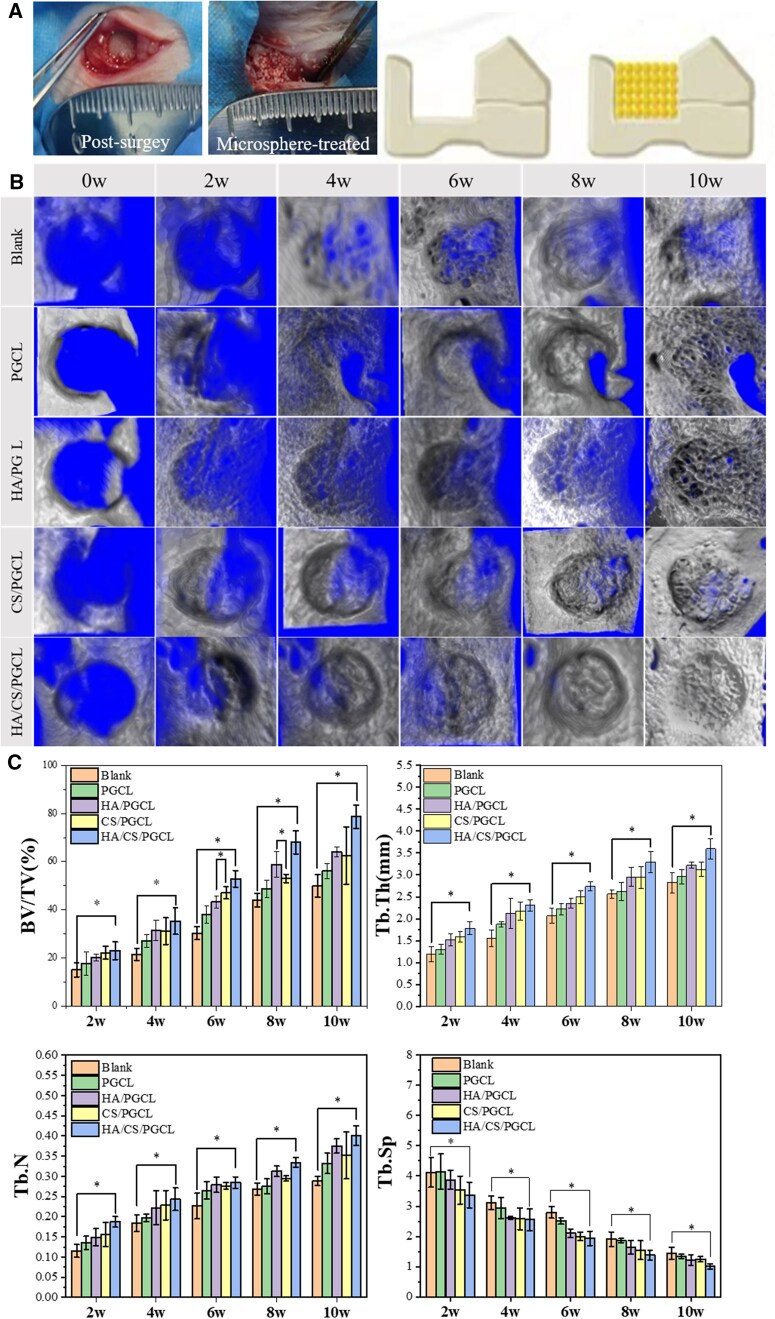
(**A**) Illustration of implanting operation. **(B**) 3D reconstructed micro-CT images of bone formation at the rabbit orbital defect areas. (**C**) The quantitative micro-CT bone parameters of bone volume/tissue volume (BV/TV) ratio, trabecular number (Tb.N), trabecular thickness (Tb.Th), trabecular separation (Tb.Sp) after 2, 4, 6, 8 and 10 weeks post-implantation. *P < 0.05.

This study visually presented the repair of rabbit orbital bone in different experimental groups at various time points (0, 2, 4, 6, 8 and 10 weeks) through CT reconstruction images (Figure 9A). At 0 weeks, all groups showed complete orbital bone defects with clear boundaries, setting a uniform starting point for observing the effects of different materials on bone repair. By week 2, minimal new bone formation was observed in the blank control group, indicating poor early repair. The PGCL group showed slight new bone formation, reflecting limited early efficacy of PGCL alone. In contrast, the HA/PGCL group displayed more new bone than PGCL, suggesting HA enhances early osteogenesis. The CS/PGCL group exhibited more prominent new bone formation, likely due to CS-derived ions or intrinsic properties. The HA/CS/PGCL group demonstrated the most significant early new bone formation, highlighting the synergistic advantage of the ternary composite. At week 4, the blank control group still had slow new bone growth, and the PGCL group’s new bone formation remained slow. The HA/PG group’s new bone formation accelerated, the CS/PGCL group’s new bone formation further sped up with a closing trend at the bottom, and the HA/CS/PGCL group had a large amount of new bone filling the defect area, again emphasizing its synergistic advantage. By 6 weeks, the blank control group’s new bone increased slightly but repair was still poor. The PGCL group showed more new bone formation, the HA/PG group continued to accumulate new bone, the CS/PGCL group had significant new bone formation with the bottom basically closed, and the HA/CS/PGCL group not only densely filled the defect with new bone but also showed good growth momentum. At week 8, the blank control group had minimal new bone growth. The PGCL group’s new bone formation accelerated, the HA/PGCL group’s new bone formation continued to rise with a growth rate exceeding the CS/PGCL group, the CS/PGCL group’s new bone formation was significant but slowed down, and the HA/CS/PGCL group had complete closure of the bone defect with abundant new bone generation, indicating nearly completed osteogenesis and good bone quality. By week 10, the blank control group had minimal new bone generation and the defect remained unclosed. The PGCL group showed little increase in new bone and repair was still unsatisfactory. The HA/PG group had stable new bone growth, the CS/PGCL group’s new bone formation remained significant, and the HA/CS/PGCL group maintained complete closure of the defect.

Further quantitative analysis (Figure 9B) of bone volume to tissue volume ratio (BV/TV), trabecular number (Tb.N), trabecular thickness (Tb.Th) and trabecular separation (Tb.Sp) revealed that over time (2, 4, 6, 8 and 10 weeks), BV/TV, Tb.Th and Tb.N values gradually increased in all groups, with BV/TV showing more significant increases over time, while Tb.S*p* values correspondingly decreased. This aligns with the physiological process of gradual bone accumulation and densification of trabecular structures during bone repair. Specifically, the Blank group showed minimal changes in these indicators throughout the experiment, confirming its poor repair ability. The PGCL group had higher indicator values than the Blank group, indicating some bone—repair potential, but still at a low level. The CS/PGCL group had higher BV/TV, Tb.Th and Tb.N values than the HA/PGCL group in the first 6 weeks, suggesting that calcium ions released from CS degradation played a dominant role in bone induction and new bone formation, consistent with *in vitro* ALP test results and osteogenic gene results. However, at 8 weeks, the HA/PGCL group surpassed, indicating that HA’s osteogenic properties became more prominent over time and played a key role in late—stage bone repair. The HA/CS/PGCL group consistently maintained high indicator values, strongly proving that the synergistic effect of CS and HA can more effectively promote bone defect repair, from initiating bone formation in the early stage to maintaining bone quality improvement in the late stage, showing excellent bone-repair effects and providing a valuable reference for future research on orbital bone defect repair materials. In summary, using both imaging and quantitative analysis, this study clearly revealed the dynamic changes and efficacy differences of different material combinations in rabbit orbital bone repair, providing solid data for material optimization and clinical application in bone defect repair.

### Histological analysis of repair area

**Figure 10:**
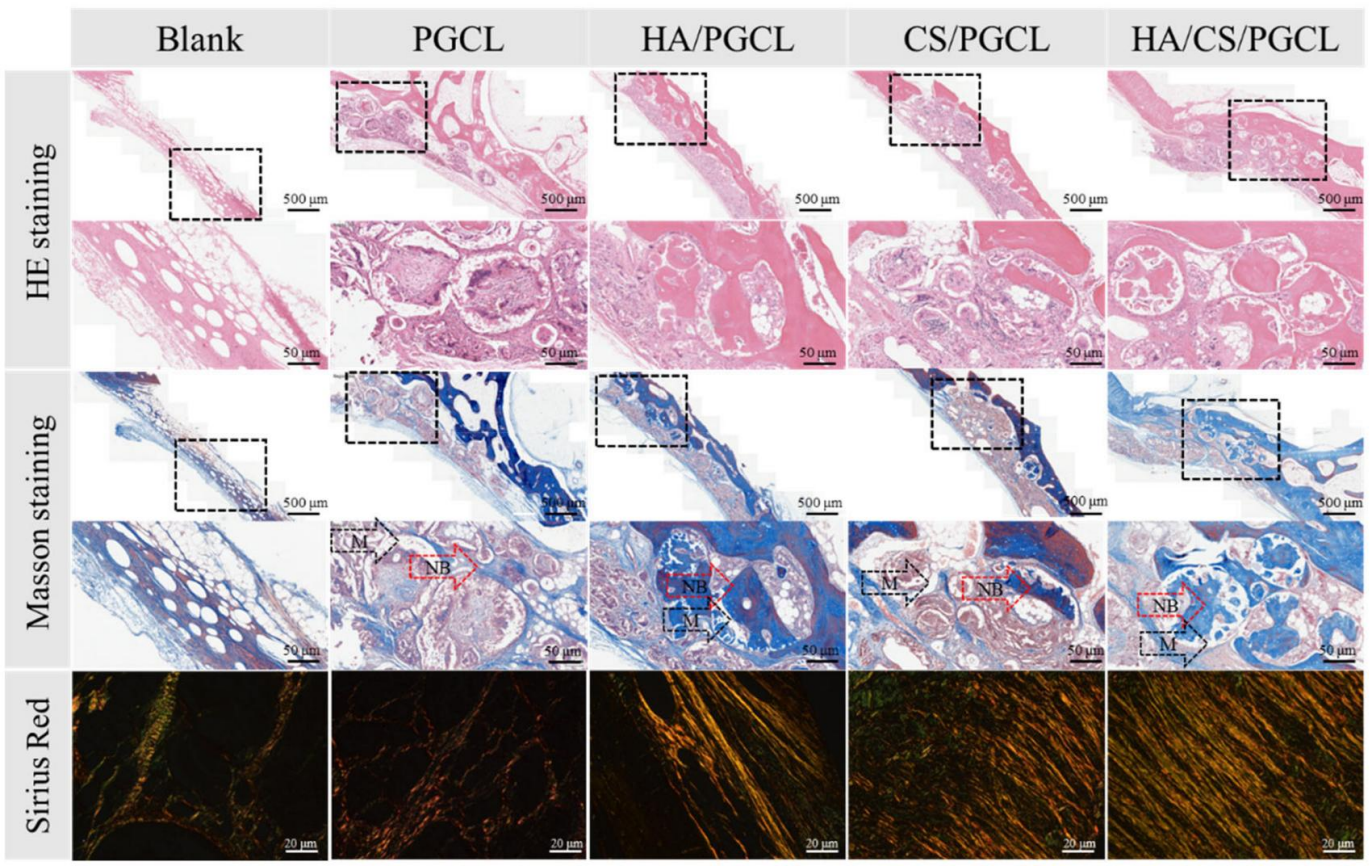
H&E staining, Masson trichrome staining and Sirus Red staining in operative site at 10 weeks. M denotes microsphere, NB denotes new bone. The scale bars are 500 μm and 50 μm, respectively.

This study comprehensively analyzed rabbit orbital bone tissue using multiple staining methods to investigate the effects of different material combinations on bone repair. Results in Figure 10 provide valuable insights for bone tissue engineering.

HE staining, a classic method for visualizing tissue structure, showed cell nuclei-stained deep blue by hematoxylin (clearly defining their distribution) and cytoplasm/matrix pink/red by eosin. The blank control group lacked significant bone formation, with fibrous tissue and undifferentiated cells dominating indicating the orbital microenvironment cannot spontaneously initiate efficient bone regeneration without inductive components, remaining in an early repair phase characterized by fibrous scarring rather than functional bone construction. The PGCL group showed modest improvement versus blank controls, with cell infiltration and matrix deposition, but insufficient bone formation. This suggests PGCL alone can recruit cells and promote matrix secretion but lacks the drive for osteogenic differentiation, failing to meet bone defect repair needs. In contrast, the HA/PGCL group exhibited enhanced bone formation: HA, a natural bone inorganic component, provided optimal cell adhesion sites, mimicked bone’s mineralized microenvironment and guided osteogenic differentiation to initiate regeneration. The HA/CS/PGCL group showed even denser, more robust bone formation likely due to CS-HA synergy optimizing the local microenvironment (via ion concentration and pH regulation), creating a favorable niche for cell growth, proliferation and differentiation, resulting in more mature, dense bone structure.

Masson staining, which distinguishes collagen and matrix, revealed minimal collagen deposition in the blank control group (consistent with delayed repair). The PGCL group showed limited, uneven collagen deposition which indicating insufficient induction and regulation, leading to disorganized collagen aggregation and ineffective bone matrix formation. The HA/PGCL group exhibited significantly increased collagen deposition, with HA promoting collagen secretion and assembly to support bone mineralization and maturation. The HA/CS/PGCL group further improved collagen uniformity: CS likely regulated collagen interactions and guided even deposition, enhancing matrix homogeneity and organization.

Sirius Red staining, which evaluates collagen alignment and matrix integrity, showed no collagen in the blank control group (confirming arrested repair). The PGCL group had disorganized collagen fibers-indicating insufficient guidance for ordered alignment and ineffective matrix integration. The HA/PGCL group showed increased collagen deposition with partial alignment, as HA promoted directional matrix structure. The HA/CS/PGCL group exhibited the most ordered collagen arrangement, with CS-HA synergy optimizing cross-linking and alignment to form a dense, functional matrix resembling physiological bone.

In summary, comparative analysis via multiple staining methods demonstrated differential material effects on bone repair. The HA/PGCL and HA/CS/PGCL groups showed superior bone formation and collagen deposition, with the latter excelling in uniform, organized collagen distribution. These results confirm HA and CS enhance repair efficiency, with their combination effectively driving bone maturation from trauma to functional structure which providing a robust basis for optimizing clinical bone defect strategies and advancing tissue engineering materials. Future research should explore HA-CS synergistic mechanisms, focusing on cellular signaling and gene regulation, to support precision material design and accelerate clinical translation.

## Conclusions

This study developed HA/CS/PGCL composite microspheres using the ESASE technique. HA and CS enhanced surface roughness, created hierarchical pores (0.6–2 nm), improved hydrophilicity (contact angle 49.7°) and boosted mechanical strength (32% higher elastic modulus) without altering microsphere size (240 μm). *In vitro* tests showed these microspheres leverage the distinct degradation rates of calcium sulfate and hydroxyapatite to achieve a sustained release of calcium ions, thereby meeting the dynamic demands of bone repair and achieved the temporal regulation. Additionally, the degradation of spherical calcium sulfate mesocrystal further increases the specific surface area of the composite microspheres, accelerating material degradation and facilitating bone ingrowth and achieved the spatial regulation. This synergistic design enables spatiotemporal regulation of the microspheres within the bone repair microenvironment. MC3T3-E1 cells exhibited 1.8× higher density and 1.6× higher ALP activity on HA/CS/PGCL microspheres compared to pure PGCL. *In vivo*, HA/CS/PGCL microspheres fully closed rabbit orbital bone defects in 10 weeks, achieving 45.2% bone volume ratio (BV/TV) and improved trabecular structure. The osteogenic performance of microspheres in orbital defects suggests potential for load-bearing or complex bone repair. Future work will optimize degradation rates, scale-up manufacturing and explore ion-cell signaling interactions for clinical translation.
